# Elaboration of a prognostic model associated with retinal hemorrhage in hemophagocytic lymphohistiocytosis

**DOI:** 10.1080/07853890.2026.2634471

**Published:** 2026-02-24

**Authors:** Huiwen Tian, Luping Wang, Yanli Hou, Zhaoyang Meng, Yu Wang, Hongyang Li

**Affiliations:** a Department of Ophthalmology, Beijing Friendship Hospital, Capital Medical University, Beijing, China; b Department of Radiology, Beijing Friendship Hospital, Capital Medical University, Beijing, China

**Keywords:** Hemophagocytic lymphohistiocytosis, retinal hemorrhage, prognostic model

## Abstract

**Background:**

This retrospective case–control study focused on retinal hemorrhage associated with hemophagocytic lymphohistiocytosis (HLH), integrating various ocular examination parameters to investigate the survival and prognostic factors in HLH patients.

**Methods:**

Of 1973 assessed patients, 104 were finally recruited and 1:1 matched into retinal hemorrhage and control groups, with the hemorrhage area further graded in the former. Demographic and hematological data were collected; central, inner, outer retinal thickness and choroidal vascularity index (CVI) were measured via optical coherence tomography (OCT). Cox proportional hazards regression models (CoxPH) were elaborated, followed by bootstrapping internal validation and external validation.

**Results:**

The hemorrhage group had significantly more intracranial lesions, lower hemoglobin, platelet count, and fibrinogen, and notably higher soluble interleukin-2 receptor (sIL-2R) and ferritin levels, along with significantly increased central inner retinal thickness,and reduced CVI. Ferritin and sIL-2R in the scattered hemorrhage group were significantly higher, and the survival time was significantly shorter than in the petechial hemorrhage group; scattered hemorrhage was predominant in moderate/high sIL-2R and ferritin subgroups. The CoxPH showed that retinal hemorrhage well predicted the survival and outcomes with a significantly higher hazard rate, and the model had high sensitivity, specificity, lower prediction error in external validation.

**Conclusion:**

This study elaborated a prognostic model in HLH, revealing that retinal hemorrhage is a hazard factor associated with increased severity and poorer prognosis in HLH patients.

## Introduction

Hemophagocytic lymphohistiocytosis (HLH), or hemophagocytic syndrome, is a life-threatening condition characterized by the inappropriate activation of lymphocytes and macrophages [1]. Notably, this condition is often precipitated by underlying factors such as infections, malignancies, and rheumatologic diseases—factors that are recognized as common triggers rather than complications of HLH [2]. HLH is categorized into two types: primary HLH, also referred to as familial HLH and secondary HLH. Primary HLH is caused by a genetic disruption affecting natural killer cells, cytotoxic T lymphocytes, and macrophages, which usually occurs in children. Secondary HLH, on the other hand, is often triggered by malignant, infectious, or autoimmune stimuli, and it is more common in adults. HLH has been a highly lethal disorder, with more than 40% of patients dying from refractory disease without timely and accurate diagnosis [3]. Initial treatment for HLH typically includes intrathecal therapy with corticosteroids and cyclosporine A. In addition, allogeneic hematopoietic stem cell transplantation has been associated with improved survival rates, particularly among pediatric patients [4].

HLH causes various systemic inflammatory symptoms and signs, including cytopenias, splenomegaly, low fibrinogen, marked elevations in inflammatory markers, and potential liver or brain injury [5]. Despite these widespread systemic effects, only a few cases of ophthalmological findings associated with HLH have been reported to date. These cases have identified multiple features, such as uveitis, orbital edema, and fundus disorders [6–8]. In a previous study, we retrospectively reviewed the presence and type of ocular abnormalities within HLH patients, discovering that posterior segment abnormalities, particularly retinal hemorrhage, were the most common ocular findings [9]. Retinal hemorrhage is not an independent disease, but a common feature of many ocular diseases and systemic diseases, which is defined as the presence of extravasated blood within the retina, bordered anteriorly by the inner limiting membrane (ILM), posteriorly by the retinal pigment epithelium (RPE) and Bruch’s membrane complex, constitutes a significant aspect of the range of pathological changes observed in the fundus. This condition can be readily identified through conventional ophthalmoscopy and non-invasive techniques like fundus photograph or optical coherence tomography (OCT), offering a high diagnostic value [10]. In this study, we aim to clarify the relationship between retinal hemorrhage and HLH progression through an integrated assessment of retina function and model elaboration, focusing on survival rate and prognostic factors in HLH patients.

## Methods

### Patients

**Figure 1.**
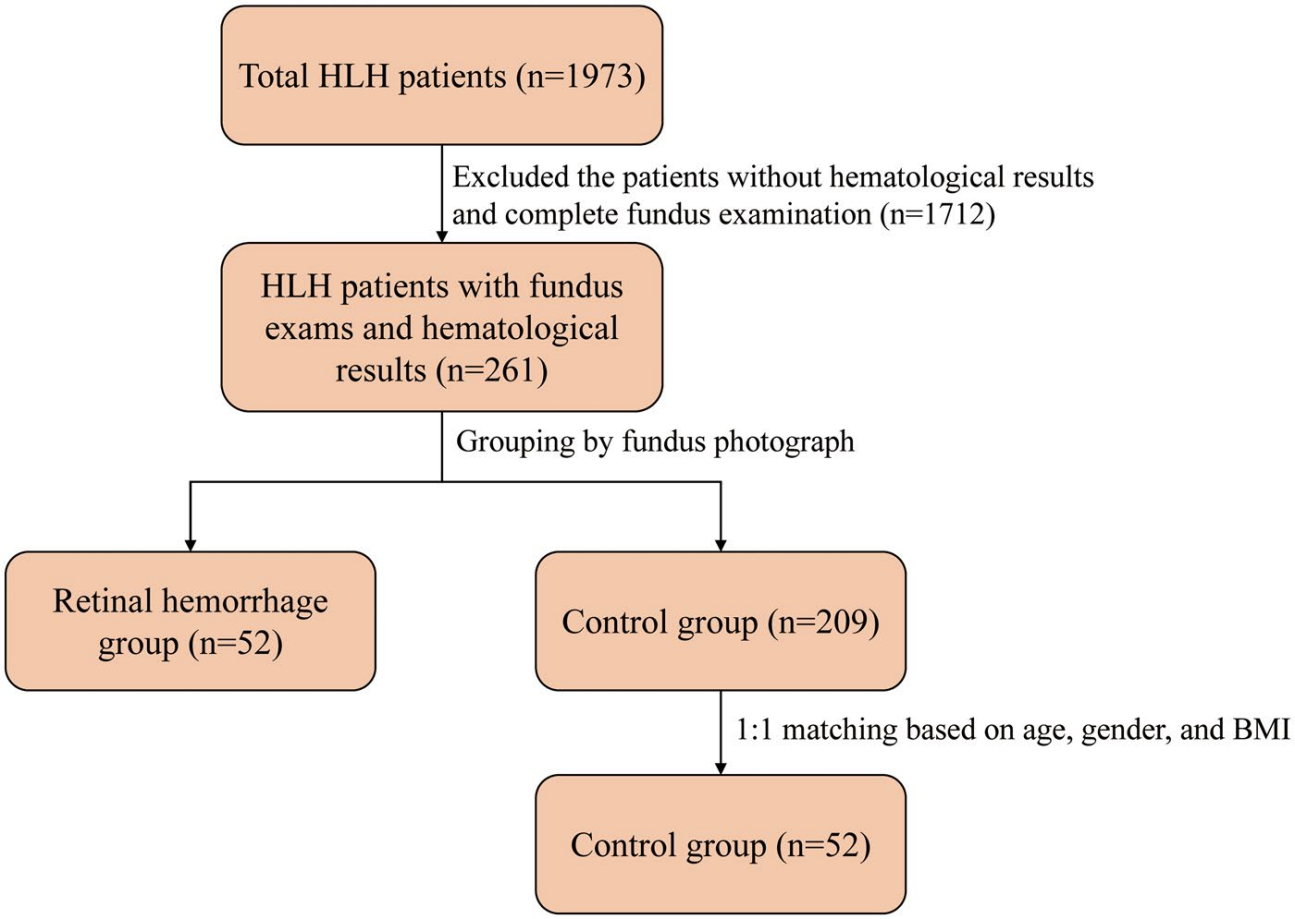
Inclusion criteria and grouping method.

This cross-sectional observational study adhered to the principles of the Declaration of Helsinki (revised in 2013), and approval was received from the ethics committee of Beijing Friendship Hospital, Capital Medical University, with approval No. 2024-P2-184. Given its retrospective nature, the written informed consent requirement was waived. A total of 1973 patients admitted to the Hematology Department of Beijing Friendship Hospital between January 2021 and July 2023 and meeting the commonly used diagnostic criteria, HLH-2004 criteria, were initially considered for inclusion. After reviewing the clinical data, these patients were excluded: patients lacking hematological results or complete fundus examination, patients for whom survival status could not be clearly determined, additionally, patients with low-quality OCT images, as well as those receiving immunosuppressive therapy or treatments for other medical conditions that could reduce platelet counts, lower hemoglobin levels, or alter ferritin levels. Notably, as this was a retrospective study, all clinical data (including survival status and follow-up information) of the 261 eligible participants were fully retrieved from the medical record system; thus, there were no patients lost to follow-up after meeting the inclusion and exclusion criteria. These were then divided into the retinal hemorrhage group (*n* = 52) and control group (*n* = 209) according to the fundus photography findings (at least one bleeding spot exists within the entire fundus). 1:1 matching during the grouping was performed based on body mass index (BMI), age, and gender, resulting in the inclusion of 104 HLH patients including 52 in the control group and 52 in the retinal hemorrhage group in the study (Figure 1). All patients had comprehensive hematological assessment during their first hospitalization, including routine blood tests, fasting venous blood glucose (FVBG), liver and kidney function tests, coagulation profile, blood lipids levels, ferritin, peripheral blood cell activity, soluble interleukin-2 receptor (sIL-2R), and enzyme expression. Additionally, each patient underwent a head CT or MRI scan upon their initial hospital admission.

### Fundus photograph and hemorrhage area grading

**Figure 2.**
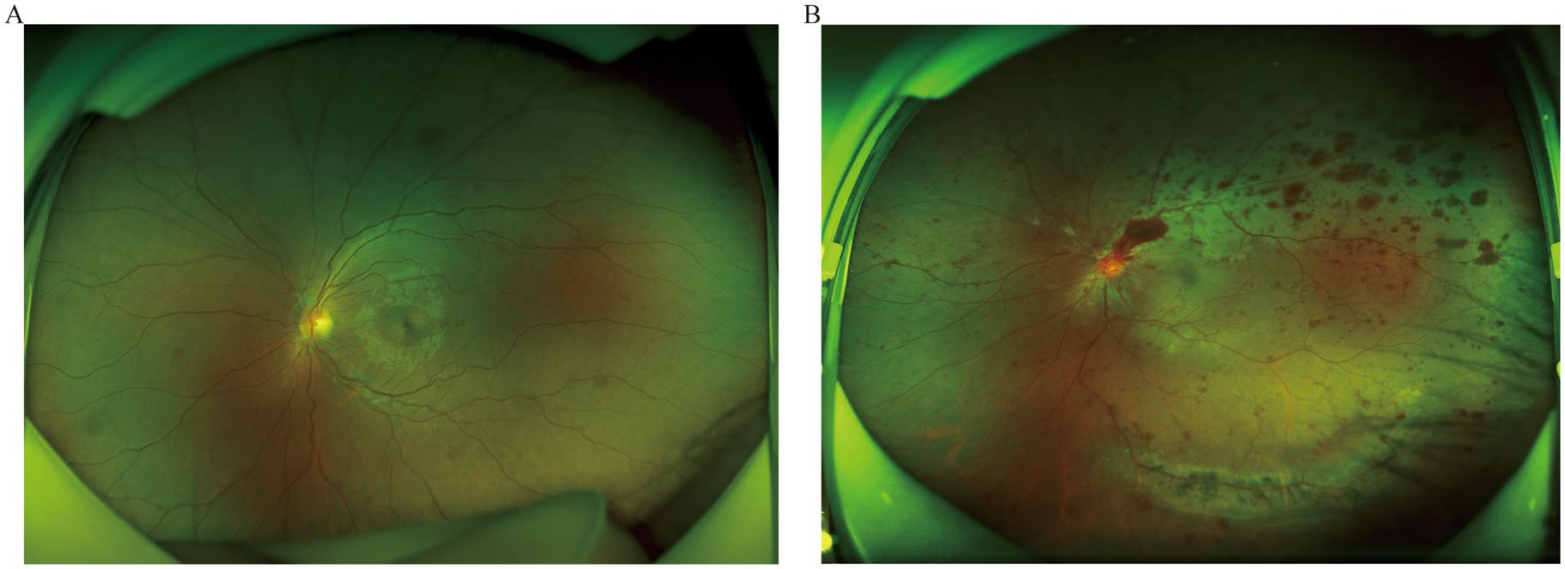
Color fundus photography of two sub-types: A, petechial hemorrhage; B, scattered hemorrhage.

Optos Daytona color fundus photography (Optos plc, Dunfermline, Scotland, UK) was used for taking color fundus photography. A medical photographer was tasked with capturing images to achieve the broadest field of view. The digital images were subsequently assessed remotely by a consultant ophthalmologist, unaware of the patient information. For patients with retinal hemorrhage, hemorrhage area was categorized into two sub-types: petechial hemorrhage (the bleeding spot was less than 1 papillary diameter) and scattered hemorrhage (Figure 2).

### Optical coherence tomography (OCT) measurements

**Figure 3.**
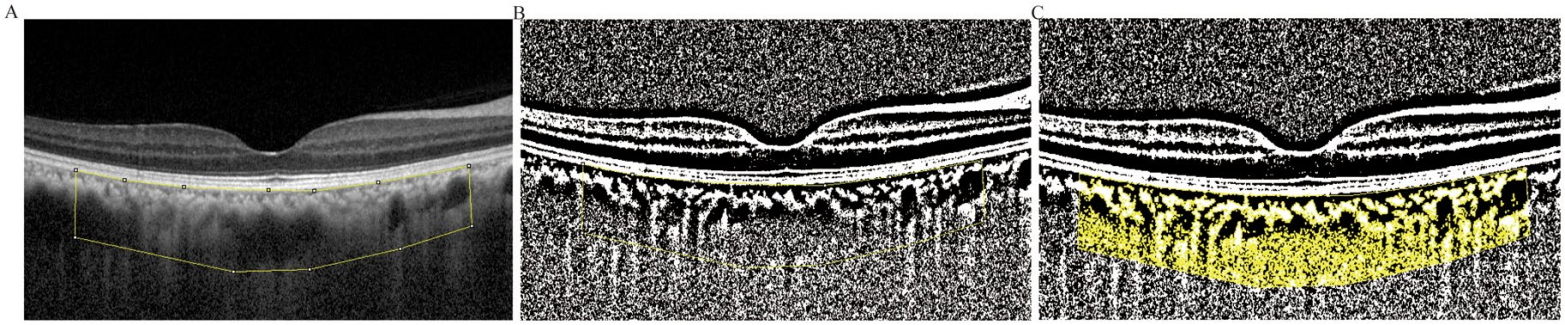
Choroidal vascularity index (CVI) measurement. A: An enhanced-deep image (EDI) was opened in Image J software and a 3-millimeters wide choroidal area was selected. B: After binarization with Niblack tool, luminal area (LA) was shown in black and stromal area (SA) was shown in white. C: LA and total cross-sectional choroidal area (TCA) was measured, CVI was calculated by LA/TCA.

A spectral-domain OCT (Spectralis, Heidelberg Engineering, Heidelberg, Germany) was used for OCT scan of all patients. The OCT examinations were conducted by the same experienced technician to ensure consistency. The central retinal thickness was automatically measured by a perpendicular line across the outer nuclear layer and retinal pigment epithelium. The outer retinal thickness was manually measured within a circular area with a diameter of 1 mm, spanning from the inner limiting membrane to the inner plexiform layer. Similarly, the inner retinal thickness measurement extended from the inner limiting membrane to the inner plexiform layer except the region without inner retinal cellular structures. The choroidal vascularity index (CVI) was assessed for all subjects using the enhanced-deep image mode, which provides an improved visualization of the choroid. To analyze the images, an enhanced-deep image (EDI) was opened in Image J software and a 3-mm wide choroidal area was selected based on references [11,12]. After binarization with Niblack tool, the luminal area (LA) and the total cross-sectional choroidal area (TCA) were measured according to established literature [13] (Figure 3). Finally, CVI was then calculated as the ratio of LA to TCA.

### Statistical analysis

The SPSS statistical software (IBM Corp., New York, United States) was used to perform statistical analyses in this study. The distribution of data was verified for normality using the Kolmogorov–Smirnov test. Depending on their distribution, data are reported as either mean ± standard deviation or median (interquartile range). Group differences were analyzed using the independent samples *t* test and the Mann–Whitney test. Overall survival was calculated from the date of the fundus examination to either the occurrence of death from any cause or the last date of follow-up (January 1, 2024). Median overall survival was calculated *via* Kaplan–Meier analysis. Cox proportional hazards regression model (Cox PH) was employed to explore the relationship between independent variables and survival outcomes using R. First, variables with relatively large original values (including sIL-2R, ferritin, and platelet) were re-scaled based on the ‘clinically significant change magnitude’ approach and multivariate Cox proportional hazards analysis was conducted. Subsequently, the dataset was split into a training set and a validation set at a 7:3 ratio, followed by the implementation of bootstrapping for internal validation and additional external validation. Statistical significance was set at a *P* value of less than 0.05.

## Results

### Patient demographic and hematological parameters

A total of 104 patients were enrolled in this investigation and were divided evenly between the retinal hemorrhage group (*n* = 52) and the control group (*n* = 52). Patient demographics and their clinical characteristics are detailed in Table 1. Due to the 1:1 matching, no significant differences in terms of gender, age, and BMI existed between the two groups. Besides, medical histories of hypertension, diabetes, FVBG and coronary heart disease (CHD) showed no significant differences between the groups. However, a statistically significant increase in intracranial lesion was observed in the retinal hemorrhage group compared to the control group (*p* = 0.008).

**Table 1.** Demographic and clinical characteristics of patients.

Variables	Retinal hemorrhage	Control	*P* value
Number of patients (n)	52	52	
Gender (male/female)	34/18	28/34	0.23
Age (years)	39.24 ± 12.78	35.88 ± 15.48	0.23
BMI (kg/m^2^)	21.34 ± 3.54	21.14 ± 3.01	0.76
Hypertension (yes/no)	7/45	3/49	0.18
Diabetes (yes/no)	3/49	7/45	0.18
FVBG	5.29 (4.53, 6.22)	4.99 (4.59, 5.79)	0.36
CHD (yes/no)	0/52	2/50	0.15
Intracranial lesion (yes/no)	25/22	14/38	0.008

BMI, body mass index; FVBG, fasting venous blood glucose; CHD, coronary heart disease.

Data are presented as mean ± standard deviation or median (interquartile range).

The comparison of hematological parameters between the two groups is shown in Table 2. Notably, the retinal hemorrhage group exhibited significantly lower levels of hemoglobin (85.98 ± 23.97 g/L), platelet count (94.10 ± 90.47 L), and fibrinogen (Fbg) (1.89 ± 0.96 g/L) than that in the control group (*p* = 0.002, *p* = 0.002, *p* = 0.04, respectively). Conversely, the levels of ferritin (1885.40 ng/mL, CI: 589.48–4924.50) and soluble sIL-2R (1885.40 pg/mL, CI: 3979.00–32566.25) were notably higher (*p* = 0.009, *p* = 0.001, respectively). No significant differences were seen in absolute neutrophil count, triglyceride, or various cell activity detection between the two groups.

**Table 2.** Comparison of hematological parameters of the retinal hemorrhage and control groups.

Variables	Retinal hemorrhage	Control	*P* value
Number of patients (*n*)	52	52	
Hemoglobin (g/L)	85.98 ± 23.97	103.52 ± 30.40	0.002
Platelet count (L)	94.10 ± 90.47	153.17 ± 100.02	0.002
Absolute neutrophil count (L)	2.99 ± 4.13	2.89 ± 2.02	0.87
Triglyceride (mmol/L)	2.15 (1.38, 3.77)	1.72 (1.34, 2.60)	0.06
Fbg (g/L)	1.89 ± 0.96	2.30 ± 1.09	0.04
NK-cell activity (%)	14.70 (12.07, 16.26)	15.81 (13.33, 17.15)	0.74
NK-cell perforin expression (%)	82.55 (74.48, 88.07)	84.94 (81.69, 92.28)	0.17
CTL perforin expression (%)	12.70 (3.00, 27.41)	20.95 (6.8, 36.49)	0.23
NK-cell granzyme B expression (%)	96.00 (91.54, 98.41)	97.81 (94.59, 98.89)	0.26
CTL granzyme B expression (%)	89.62 (65.53, 96.93)	96.21 (81.57, 98.91)	0.14
Ferritin (ng/mL)	1885.40 (589.48, 4924.50)	629.00 (203.10, 1824.00)	0.009
sIL-2R (pg/mL)	11349.00 (3979.00, 32566.25)	4397.00 (1853.00, 9891.00)	0.001

Fbg, fibrinogen; NK, natural killer; CTL, cytotoxic T lymphocyte; sIL-2R, soluble interleukin-2 receptor.

Data are reported as either mean ± standard deviation or median (interquartile range).

### Comparison of fundus parameters in HLH patients

An initial comparison of fundus parameters between the retinal hemorrhage group and the control group is summarized in Table 3. This analysis revealed a significant increase in central retinal thickness (290.44 ± 225.39 μm) and inner retinal thickness (104.27 ± 92.01 μm) among hemorrhage HLH patients (*p* = 0.02, *p* = 0.01, respectively). However, there was no significant difference in outer retinal thickness between the two groups. Additionally, the retinal hemorrhage group exhibited a notable reduction in CVI (0.635%, CI: 0.602–0.661) (*p* = 0.002).

**Table 3.** Comparison of fundus parameters of the retinal hemorrhage and control groups.

Variables	Retinal hemorrhage	Control	*P* value
Number of patients (*n*)	52	52	
Central retinal thickness (μm)	290.44 ± 225.39	217.80 ± 19.92	0.02
Inner retinal thickness (μm)	104.27 ± 92.01	69.60 ± 11.56	0.01
Outer retinal thickness (μm)	235.92 ± 59.30	220.69 ± 14.65	0.08
CVI (%)	0.635 (0.602, 0.661)	0.672 (0.637, 0.703)	0.002

CVI, choroidal vascularity index.

Data are presented as mean ± standard deviation.

To better elucidate the relationship between retinal hemorrhage and HLH, patients with hemorrhage were further divided into two sub-types based on the area of hemorrhage named petechial hemorrhage and scattered hemorrhage (*n* = 23, *n* = 20 respectively), as shown in Table 4. A comprehensive comparison of fundus and hematology was conducted between these sub-types. While there were no significant differences in fundus parameters, hemoglobin and platelet count, meanwhile, levels of ferritin (1358.10 ng/mL, CI: 388.70–2537.80) and sIL-2R (21092.00 pg/mL, CI: 10949.75–42411.50) were significantly higher in the scattered hemorrhage subgroup compared to the petechial hemorrhage subgroup (*p* = 0.01, *p* = 0.008, respectively).

**Table 4.** Comparison of fundus and hematological parameters in different hemorrhage area groups.

Variables	Petechial hemorrhage	Scattered hemorrhage	*P* value
Number of patients (*n*)	32	20	
Central retinal thickness (μm)	299.23 ± 270.01	280.70 ± 143.88	0.78
Inner retinal thickness (μm)	94.42 ± 73.35	121.20 ± 117.08	0.32
Outer retinal thickness (μm)	229.97 ± 28.31	246.40 ± 89.26	0.34
Hemoglobin (g/L)	90.07 ± 19.26	79.35 ± 29.43	0.28
Platelet count (L)	105.74 ± 94.23	77.42 ± 82.58	0.12
Ferritin (ng/mL)	1358.10 (388.70, 2537.80)	2767.00 (1105.88, 9516.85)	0.01
sIL-2R (pg/mL)	7749.50 (2483.00, 16277.25)	21092.00 (10949.75,42411.50)	0.008
Survival time (*d*)	476.0 (300.5, 651.5)	55.0 (25.41, 84.59)	<0.001

Data are reported as either mean ± standard deviation or median (interquartile range); sIL-2R, soluble interleukin-2 receptor.

We further categorized ferritin into three subgroups (low: <5000 ng/mL, moderate: 5000–15000 ng/mL, high: >15000 ng/mL) for analysis (*n* = 41, 6, 5 respectively). Cross-tabulation showed that petechial hemorrhage dominated in the low ferritin subgroup (73.2%, 30/41), while scattered hemorrhage was prevalent in moderate (83.3%, 5/6) and high (80.0%, 4/5) subgroups. Chi-square tests confirmed a significant association between ferritin subgroups and hemorrhage type (Pearson χ^2^ = 11.093, *df* = 2, *p* = 0.004; linear-by-linear association χ^2^=9.215, *df* = 1, *p* = 0.002) (Supplementary Table 1). Similarly, sIL-2R was divided into three subgroups (low: ≤10000 pg/mL, moderate: 10001–30000 pg/mL, high: >30000 pg/mL) (*n* = 23, 16, 13 respectively). Cross-tabulation and chi-square tests revealed scattered hemorrhage predominated in the high sIL-2R subgroup (69.2%, 9/13), with a significant association between sIL-2R subgroups and hemorrhage type (Pearson χ^2^ = 9.703, *df* = 2, *p* = 0.008; linear-by-linear association χ^2^ = 9.516, *df* = 1, *p* = 0.002) (Supplementary Table 2).

Furthermore, Kaplan–Meier survival analysis revealed significant differences in survival time between two groups (χ^2^ = 15.799, degrees of freedom = 1, *p* < 0.001). The median survival time in petechial hemorrhage subgroup was 476.0 days (95% CI: 300.5–651.5 days), while the median survival time was 55.0 days (95% CI: 25.41–84.59 days) in scattered hemorrhage subgroup.

### The elaboration and internal validation of Cox PH in HLH

**Figure 4.**
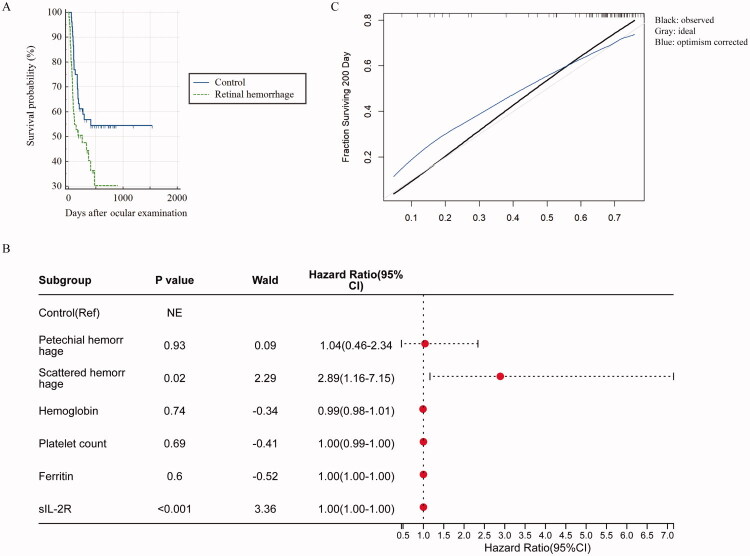
A: Kaplan–Meier survival curves for the retinal hemorrhage group and control group. B: A forest plot about Cox proportional hazards regression model (Cox PH). C: A calibration plot for predicting the fraction of subjects surviving 200 days. Black line for reference, red line for model.

To assess the impact of retinal hemorrhage on the survival probability of patients with HLH, a Kaplan–Meier survival analysis was performed. The survival curves of the retinal hemorrhage (388.96 ± 60.84 days) and control groups (904.29 ± 98.54 days) are shown in Figure 4A. Analysis of these curves demonstrated that the presence of retinal hemorrhage significantly decreased survival probability among HLH patients (χ^2^ = 4.942, degrees of freedom = 1, *p* = 0.03).

Next, the original data of sIL-2R, ferritin, and platelet were transformed in accordance with the ‘clinically significant change magnitude’ scaling protocol, with the scaling units defined as ‘per 10,000 pg/mL increase’, ‘per 1,000 ng/mL increase’, and ‘per 50 × 10^9^/L decrease’ respectively and CoxPH was elaborated. The forest plot show that retinal hemorrhage (both scattered hemorrhage and petechial hemorrhage) has a significantly higher hazard rate while per 10,000 U/mL increase of sIL-2R has a relatively small decreased in the hazard rate compared to the control (*p* = 0.001, *p* = 0.001, *p* = 0.001) (Figure 4B). Table 5 presents Bootstrap validation for the Cox PH, which show that the model performs reasonably well after correction with low prediction error and reduced overfitting (Table 5). The Harrell’s concordance index was 0.66 suggests the model has moderate discriminative ability, meanwhile, calibration plot for predicting the fraction of subjects surviving 200 days suggesting the model has reasonable calibration (Figure 4C).

**Table 5.** Bootstrap validation and model validation metrics.

Index	Original value (index.orig)	Training set	Test set	Optimism	Corrected value (index.corrected)	Sample size (*n*)
Dxy	0.3254	0.4023	0.2825	0.1198	0.2056	198
*R*²	0.2940	0.3525	0.2315	0.1210	0.1730	198
Slope	1.0000	1.0000	0.7667	0.2333	0.7667	198
*D*	0.0858	0.1114	0.0645	0.0469	0.0389	198
*U*	−0.0073	−0.0074	0.0206	−0.0280	0.0207	198
*Q*	0.0931	0.1189	0.0439	0.0750	0.0182	198
*G*	0.9109	1.1909	0.8859	0.3050	0.6059	198

D, discrimination; U, Unreliability; Q, Calibration; Dxy, Discrimination Index based on the predictive probability and outcome; g, goodness-of-fit statistic.

### The external validation of Cox PH in HLH

**Figure 5.**
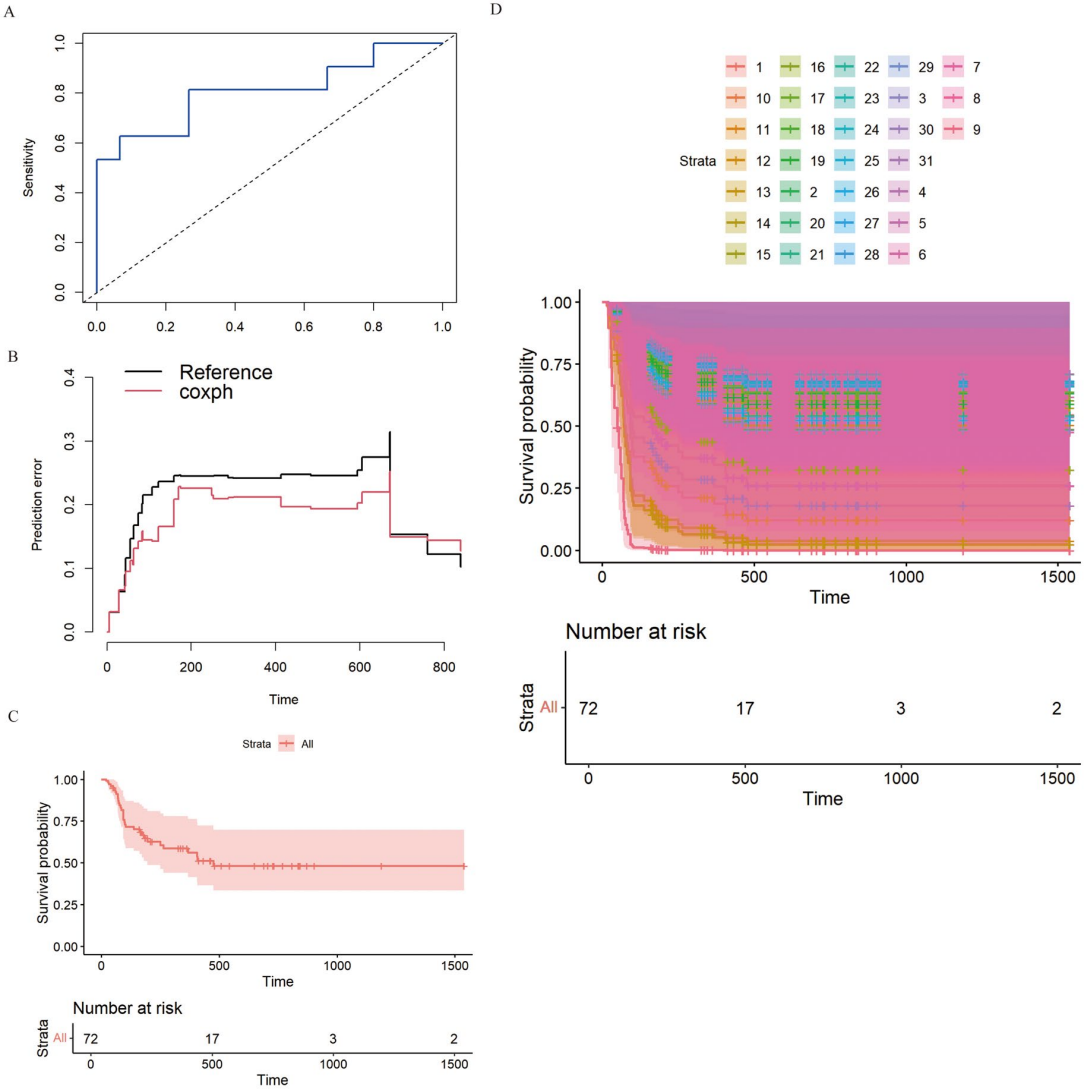
The external validation of The external validation of Cox PH in HLH (Cox PH). A: Time-dependent ROC curves of the validation set. B: Brier score of the Cox model (red) and reference benchmark (black). C: Kaplan–Meier survival analysis for training set. D: Kaplan–Meier survival analysis for external validation set.

The validation set was used to assesses the model’s performance using time-dependent ROC curves and Brier score (Figure 5A and B). The ROC curves hug the top-left, indicating high sensitivity and high specificity. Moreover, the Cox model (red) has lower prediction error and better prediction performance over time than the reference benchmark according to Brier score. Next, we quantified the average contribution of each covariate to the model’s predictions in the external dataset and the result show that retinal hemorrhage has the largest positive impact on predicted risk (with covariate contributes to +0.2486). Kaplan–Meier survival analysis was performed again for a training set and an external validation set (Figure 5C and D). In detail, for training set, a sharp decline in survival probability (from 1.0 to ∼0.5) was shown at early stage and survival probability stabilized at 0.5 at last. The number at risk starts with 72 subjects at time 0, drops to 17 at 500, 3 at 1000, and 2 at 1500. For external validation set, clear separation between subgroups at early stage (0–500 time units) and stabilize at distinct survival levels at late stage (>500 time units).

## Discussion

This study provides insight into the retina functional evaluation and clinical characteristics of HLH in a Chinese population, elaborated a prognostic model associated with retinal hemorrhage. Our findings indicate that retinal hemorrhage is not only a marker of fundus abnormalities but also correlates significantly with the hematological parameters in HLH patients. Importantly, retinal hemorrhage show as a prognostic model of HLH through retina functional analyzation, emerging as a sensitive indicator of an increased risk of mortality in these patients.

OCT offers high-resolution insights into the blood flow of the retina and choroid. We noted a marked increase in central and inner retinal thickness among hemorrhage patients, while outer retinal thickness remained unchanged. A plausible explanation is that intraretinal hemorrhage induces retinal edema, primarily thickening the inner retina. The inner retinal layer, essential for visual function, comprises a network of retinal interneurons, including bipolar cells, amacrine cells, and horizontal cells [14]. However, a notable limitation of this study is the lack of longitudinal data on retinal hemorrhage reversibility in HLH survivors, leaving uncertainty about whether hemorrhages resolve over time or persist as irreversible changes (e.g. residual retinal scarring).

To address this gap and extend the study’s value, future research should prioritize three key directions. First, conduct multicenter, large-sample longitudinal follow-up studies to track dynamic changes in retinal hemorrhage in surviving HLH patients. Focus on analyzing associations between hematological thresholds (e.g. platelet count >50 × 10^9^/L, hemoglobin >90 g/L) and hemorrhage reversibility, identify clinical intervention windows for different recovery patterns, and inform targeted ocular rehabilitation protocols. Second, explore the link between underlying etiologies of secondary HLH and retinal hemorrhage risk: stratify patients with high hemorrhage hazard ratios (HR) by etiology (e.g. viral infection-, lymphoma-, or autoimmune-associated HLH), compare HLH severity indicators (e.g. fever duration, organ failure count) across subgroups, and clarify whether the hemorrhage-severity association and prognostic value of hemorrhage vary by cause—this will enable etiology-specific risk assessment. Third, integrate data on anticoagulant use (e.g. low-molecular-weight heparin) and ocular neovascularization-associated agents (e.g. anti-VEGF inhibitors), including dosage, duration, and timing relative to hemorrhage onset. Use multivariate regression or propensity score matching to adjust for these confounders, verify the robustness of associations between HLH-specific markers (e.g. ferritin, sIL-2R) and hemorrhage, and quantify the independent contribution of drugs to bleeding risk—strengthening causal inference and guiding medication safety.

In addition, a notable decrease in CVI was observed in patients with retinal hemorrhage. CVI is crucial for assessing structural and vascular changes in the choroid as potential indicators of retinal damage. Indeed, Impairment of the choroidal circulation invariably results in diminished intraretinal vascular flow and subsequent damage to the retinal photoreceptors [11]. A decline in CVI reflects reduced choroidal blood flow, suggesting ischemic changes in the choroid and retina of HLH patients. This supports the hypothesis that vitreous hemorrhage in HLH may stem from retinal ischemia caused by microcirculatory disorders, leading to neovascularization and subsequent rupture. Our study aligns with previous research indicating an increase in CVI following remission in acute leukemia, as well as reports of subclinical choroidal involvement in patients with malignant hematological diseases. This consistency reinforces the notion that HLH adversely affects the fundus and choroidal functions, suggesting a broader impact of the disease on ocular health [15,16].

The coagulation system plays a pivotal role in the occurrence of retinal hemorrhage in HLH patients, as evidenced by our observations. A mild decrease in hemoglobin, platelet count, and fibrinogen was observed, along with a significant increase in ferritin levels in patients experiencing hemorrhage. This is consistent with the findings of Goel et al. who reported a case of retinal hemorrhages at presentation in a patient with idiopathic thrombocytopenic, accompanied by thrombocytopenia and anemia [17]. Hypofibrinogenemia has been identified as the most commonly reported coagulation anomaly in HLH, closely linked to higher mortality rates, and considered a part of the diagnostic criteria for HLH [18]. The presence of hypofibrinogenemia significantly elevates the risk of major hemorrhage events and mortality [19]. These findings underscore the critical impact of coagulation system dysfunctions on the fundus function, reinforcing the association between retinal hemorrhage and an adverse prognosis in HLH patients.

Ferritin and sIL-2R are two crucial biomarkers included in the diagnostic criteria according to the HLH-2004 diagnostic guidelines [20], and their elevation has been observed in HLH patients experiencing vitreous hemorrhage, as determined through hematology examination. Moreover, our study has established a correlation between these biomarkers and the extent of hemorrhage, with significantly higher increases observed in the scattered hemorrhage group compared to the petechial hemorrhage group. Ferritin is the primary storage form of iron in the human body, and plays a regulatory role in calming immune response under normal physiologic conditions by inhibiting the granulocyte number, lymphocyte division, bacterial growth, and reactive oxygen species related cell damage. In HLH characterized by uncontrolled cytotoxic lymphocyte proliferation, hepatic injury releases ferritin as an acute phase reactant [21]. Previous studies showed that hyperferritinemia can lead to retinal ganglion cell death and permanent vision damage [22]. In addition, another study demonstrated that iron-loaded ferritin accumulates in lysosomes and is released into the extracellular space in lysosomal membrane-enclosed vesicles, contributing to the death of retinal pigmented epithelial cells. Elevated levels of sIL-2R signal an ongoing immune response, serving as a marker for monitoring immune-mediated diseases. Several studies have identified high sIL-2R expression in cases of autoimmune uveitis, and treatments targeting sIL-2R have been shown to reduce inflammation [23,24]. Our results indicate that sIL-2R, as a closely associated yet relatively low-risk critical component in the HLH prediction model, plays a significant role in predicting the mortality of patients with HLH. While direct studies linking sIL-2R levels to retinal hemorrhage are currently lacking, our observations suggest a possible association with sIL-2R mediated inflammatory pathways within the retina, meriting further investigation.

HLH is a formidable hematological syndrome characterized by a relatively low incidence but high mortality rates, with reported mortality varying significantly from 20.4% to 88% across different studies, influenced by population demographics and the duration of follow-up [25]. Particularly in adult patients in ICUs, HLH is linked to an elevated mortality rate, underscoring the urgency for prognostic factors that facilitate rapid diagnosis and potentially improve outcomes [26]. Retinal hemorrhage, emerges as a hazard factor adversely affecting the survival time and probability of patients with HLH in our study. The prognostic model related with retinal hemorrhage can reasonably distinguish between subjects with different survival prospects within the training set, which support the model’s potential for practical application in predicting survival outcomes, particularly in clinical settings where risk stratification of new patients is needed. Previous reports have indicated that retinal hemorrhage is correlated with various systemic changes such as diabetic retinopathy, autoimmune disease, and carcinoma [27–30]. In the field of hematological disease, Guillaume et al. reported the association between retinal hemorrhage and autoimmune hemolytic anemia, while others reported that unilateral vitreous hemorrhage is an initial presentation for chronic myelogenous leukemia [31,32]. A specific study focusing on idiopathic aplastic anemia within an Asian population found that 37% of subjects presented with retinal hemorrhage [33]. The retina’s normal function depends on adequate blood cell activity to transport oxygen and clear metabolic waste. However, in HLH, the abnormal activation of the phagocytic system significantly reduces the normal function of the hematopoietic system. In order to compensate for the reduced oxygen-carrying capacity, retinal venous system adaptations occur, including turbulent and enhanced flow, placing stress on the endothelium through ischemia, dilatation, and turbulence, which ultimately leads to retinal hemorrhage.

To our knowledge, this is the first study to establish a prognostic model specifically targeting the ocular manifestations of HLH, marking a fundamental distinction from prior research on HLH-related retinal hemorrhage. While previous studies merely confirmed retinal hemorrhage as a common ocular finding in HLH, we advance this field by demonstrating through Cox proportional hazards regression that retinal hemorrhage is an independent risk factor for mortality in HLH [34,35]. This finding transforms retinal hemorrhage from a ‘descriptive clinical sign’ into a ‘clinically actionable prognostic tool’ for risk stratification in HLH management.

Furthermore, we introduce a novel hemorrhage subtype stratification (petechial vs. scattered hemorrhage) and clarify its prognostic significance: unlike prior descriptive studies that treated retinal hemorrhage as a homogeneous entity, our refinement reveals that not all retinal hemorrhages confer equal prognostic risk. We also integrate ocular structural parameters (retinal thickness, CVI) with systemic hematological markers (ferritin, sIL-2R) to construct a multi-dimensional prognostic model, which comprehensively reflects the interplay between ocular and systemic pathologies in HLH—an integration absent in previous studies that focused solely on either domain [36]. Finally, the model undergoes robust dual validation (bootstrapping internal validation and external cohort validation), ensuring its reliability and clinical applicability, which further distinguishes it from prior observational studies lacking predictive modeling or systematic validation.

Although there are certain limitations in the establishment of the model in this study, such as a relatively small sample size, our internal validation initially suggested potential overfitting—likely attributed to the training set’s moderate sample size, which may have led the model to partially ‘learn’ noise (e.g. sporadic extreme values of laboratory indicators like sIL-2R) rather than generalizable prognostic patterns. Notably, this overfitting signal was not reflected in external validation: the model exhibited excellent performance in the independent external cohort, which we attribute to two key factors. First, the external validation was conducted using a genuine independent cohort without sampling bias, which can better reflect the model’s true generalization ability, thus outperforming the ‘biased estimates’ from internal validation [37]. Second, the core predictive role of retinal hemorrhage—consistently linked to poor survival in both cohorts—was more stably captured in the external validation, as the cohort lacked the rare ‘retinal hemorrhage with favorable outcome’ cases that had slightly skewed the training set.

As a retrospective study, we acknowledge potential data completeness and accuracy limitations common to this design. To mitigate these, we implemented targeted measures: strict inclusion/exclusion criteria to exclude patients with missing key data, standardized fundus/OCT measurement protocols (by experienced staff, masked review) to reduce bias, cross-validation of hematological indicators (e.g. ferritin, sIL-2R) across sources, and exclusion of confounder-related cases. Bootstrapping internal validation and robust external validation (high sensitivity/specificity, low prediction error) further confirm model reliability. Future prospective studies with standardized data collection will enhance generalizability.

This study has undergone sufficient internal and external validations, which concluded that retinal hemorrhage is important and reasonable as a component of the prediction model for HLH. Moreover, to our knowledge, this model is the first prognostic model established so far specifically targeting the ocular manifestations of HLH. We believe this study can provide assistance for the subsequent early diagnosis of HLH.

## Conclusion

Our study elaborated a prognostic model associated with retinal hemorrhage in HLH, elucidates that retinal hemorrhage significantly contributes to the severity and adversely affects the prognosis of patients with HLH, marking a pivotal advancement in our understanding of the ocular manifestations of this severe systemic condition. By facilitating early identification and intervention for such complications, our study aims to raise awareness amongst both ophthalmologists and haematologists, guiding early treatment and ultimately aspiring to improve patient outcomes.

## Supplementary Material

SupTable 2.docx

SupTable 1.docx

## Data Availability

The data that support the findings of this study are available from the corresponding author, Hongyang Li, upon reasonable request.

## Abbreviations

HLH: hemophagocytic lymphohistiocytosis
ILM: inner limiting membrane
OCT: optical coherence tomography
BMI: body mass index
sIL-2R: soluble interleukin-2 receptor
CVI: choroidal vascularity index
LA: luminal area
TCA: total cross-sectional choroidal area
CHD: coronary heart disease
