# Supplementary material for: Elaboration of a prognostic model associated with retinal hemorrhage in hemophagocytic lymphohistiocytosis
Source: Ann Med. 2026 Feb 24;58(1):2634471. doi: 10.1080/07853890.2026.2634471 (PMC12943805; doi:10.1080/07853890.2026.2634471)
Supplement: SupTable 2.docx [file IANN_A_2634471_SM9009.docx]

Supplementary Table 2: Chi-square test results for sIL-2R subgroups versus retinal hemorrhage type

| Test type |  | Value | DF | Asymptotic Significance |
| --- | --- | --- | --- | --- |
| Pearson Chi-Square |  | 9.703 | 2 | 0.008 |
| Likelihood ratio |  | 10.061 | 2 | 0.007 |
| Linear-by-linear association |  | 9.516 | 1 | 0.002 |
| Valid cases |  | 52 | - | - |

sIL-2R, soluble interleukin-2 receptor; DF, degrees of freedom.
